# Official or Officinal?

**Published:** 1878-09

**Authors:** 


Official or Officinal?—We are loth to suddenly part company with old friends, be they human, trophies, theories or words that may have become endeared to us by association or usage, yet this tie should never be so strong as to lead to the prejudicial side. Whatever is correct should be adopted and used, even though its discovery was not given to the fathers, but left to the sons. The adoption of *official* will be doing a justice to our language by giving to the word its rightful place, so long falsely occupied by officinal.

The term *officinal* is in favor with many because of its long usage; no thought being taken of its correctness or incorrectness.

No doubt the botanical use of officinal as a specific name for plants used in medicine, as althæa *officinalis*, zingiber *officinale*, etc., has been one of the chief means of giving the word its prominence with the medical and pharmacal fraternities, but in giving specific names to plants already much used, and to be had in the apothecaries' shops, botanists meant merely that the particular althæa or zingiber should thereafter be known, for distinction only, as the species "of the shops," or officinal, the word having no reference to authority. Now, medicines that are recommended to be used by such bodies as the National Pharmacopœial Convention, British Council, etc., are *official*—given under authority—in the countries over which they have jurisdiction.

It is true that both words are derived from the same root, but officinal is the older, coming to us directly from the Latin *officina*, a shop; while official, the younger, comes to us through the French *officiel*.

"An *official* formula is one given under authority. An *officinal* formula is one made in obedience to the customary usage of the shop (*officina*). To state that any preparation under the sanction of the Pharmacopœia is officinal, is a misapprehension of the meaning of the word."—*Brough.*

"The Pharmacopœia and all in it are official (*office*, Fr. from L. *officium*, an office). There are many things which, in pharmacy, are officinal (Fr. from L. *officina*, a shop) but not official. To restrict the word *officinal* to the contents of a pharmacist's shop, and to that portion of the contents which is pharmacopœial, is radically wrong, and should be avoided."—*Note to Attfield's Chemistry, 5th Ed., p.* 25.

It has been objected that the "innovation," as it is called, has no where received support. In refutation of this I note that it has already been adopted by Professor Attfield, in his "Chemistry;" Mr. Squire, in his "Companion to the British Pharmacopæia;" Mr. Wills—the most successful teacher of preliminary pharmacy in England—in the Westminster College of Chemistry and Pharmacy, and in our own country by the United States Marine Hospital Service. This ought surely to be authority sufficient to warrant the luke-warm in deciding in its favor, since the outlook is so bright.

Official has another advantage; it is one syllable shorter than the old word.

The objection that the word is new cannot obtain, because it long has been, and is in daily use in governmental circles. It is only taking a new direction.

The customary use of the term *unofficinal* is radically wrong—its true meaning being that anything that is unofficial is not to be had in the shops; while many articles that have never been accredited a place in any Pharmacopœia, and others that have been expugned, are constantly kept on sale in the shops. This ambiguity will cease to exist with the adoption of the term *unofficial*, which has but one meaning in medicine: not recognized by national authority.

In view of all this, I beg authors that are about to issue books that may be used as authority, and the Pharmacopœial Convention of the Sixth Revision, to note the term and adopt it, thereby accepting the inevitable.

I hope we may not be given an opportunity to say, with Job, "They have refused to receive correction."—*American Journal of Pharmacy.*

